# Evolutionary convergence in the biosyntheses of the imidazole moieties of histidine and purines

**DOI:** 10.1371/journal.pone.0196349

**Published:** 2018-04-26

**Authors:** Alberto Vázquez-Salazar, Arturo Becerra, Antonio Lazcano

**Affiliations:** 1 Facultad de Ciencias, Universidad Nacional Autónoma de México, Cd. Universitaria, Cd. de México, México; 2 Miembro de El Colegio Nacional, Centro, Cd. de México, México; Weizmann Institute of Science, ISRAEL

## Abstract

**Background:**

The imidazole group is an ubiquitous chemical motif present in several key types of biomolecules. It is a structural moiety of purines, and plays a central role in biological catalysis as part of the side-chain of histidine, the amino acid most frequently found in the catalytic site of enzymes. Histidine biosynthesis starts with both ATP and the pentose phosphoribosyl pyrophosphate (PRPP), which is also the precursor for the *de novo* synthesis of purines. These two anabolic pathways are also connected by the imidazole intermediate 5-aminoimidazole-4-carboxamide ribotide (AICAR), which is synthesized in both routes but used only in purine biosynthesis. Rather surprisingly, the imidazole moieties of histidine and purines are synthesized by different, non-homologous enzymes. As discussed here, this phenomenon can be understood as a case of functional molecular convergence.

**Results:**

In this work, we analyze these polyphyletic processes and argue that the independent origin of the corresponding enzymes is best explained by the differences in the function of each of the molecules to which the imidazole moiety is attached. Since the imidazole present in histidine is a catalytic moiety, its chemical arrangement allows it to act as an acid or a base. On the contrary, the *de novo* biosynthesis of purines starts with an activated ribose and all the successive intermediates are ribotides, with the key β-glycosidic bondage joining the ribose and the imidazole moiety. This prevents purine ribonucleotides to exhibit any imidazole-dependent catalytic activity, and may have been the critical trait for the evolution of two separate imidazole-synthesizing-enzymes. We also suggest that, in evolutionary terms, the biosynthesis of purines predated that of histidine.

**Conclusions:**

As reviewed here, other biosynthetic routes for imidazole molecules are also found in extant metabolism, including the autocatalytic cyclization that occurs during the formation of creatinine from creatine phosphate, as well as the internal cyclization of the Ala-Ser-Gly motif of some members of the ammonia-lyase and aminomutase families, that lead to the MIO cofactor. The diversity of imidazole-synthesizing pathways highlights the biological significance of this key chemical group, whose biosyntheses evolved independently several times.

## Introduction

**Fig 1:**
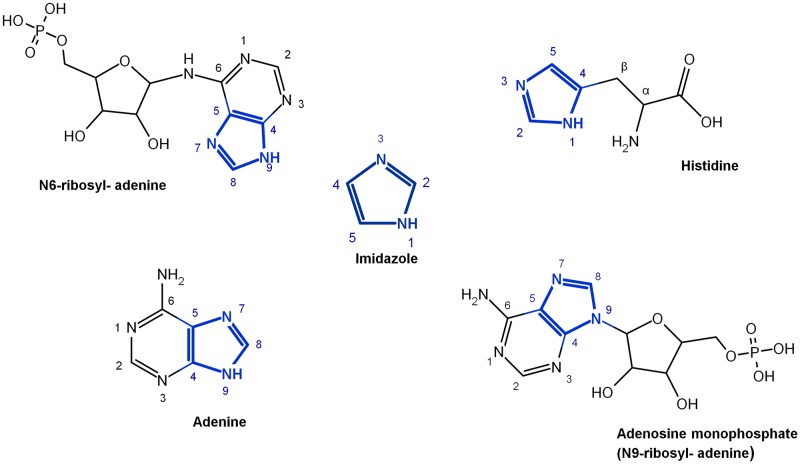
Structure of imidazole and imidazole-derivatives with atom numbering. The unsubstituted imidazole group is shown in the center. Other important imidazole molecules are shown: histidine (2-amino-3-(1H-imidazol-4-yl), adenine (9H–purin-6-amine), adenosine monophosphate (N9-ribosyl-adenine), and N6-ribosyladenine (which synthesis has been reported in prebiotic chemistry experiments).

The imidazole group is a five-membered heterocyclic chemical compound containing a tertiary nitrogen and an imino group. It is present in the structure of several key molecules of major biological significance, most notably purines and histidine. The structure of the unsubstituted imidazole ring has two nitrogen atoms in positions 1 and 3 (also named pyrrole- and pyridine-nitrogen atoms, respectively) (Fig 1). The pyrrole nitrogen is a very weak acid with a pK_a_ = 14.4. In contrast, the pK_a_ of the pyridine nitrogen is 6.9, which allows it to accept a proton under neutral pH conditions [1]. This phenomenon does not occur in the imidazole moiety of purines, but it takes place in the imidazole side-chain of histidine, whose pK_a_ value is in the range of 6 to 7 as a result of the electron-withdrawing inductive effect of the protonated amino group [2,3]. Bioinformatic analyses have shown that histidine is the most frequently found catalytic residue in the active sites of enzymes [4]. This phenomenon may be explained by the level of ionization of the imidazole moiety of histidine, which is estimated to be between 9–50%, resulting in its well-known ability to act as a general acid or base to donate or accept protons during a chemical reaction. This property, together with the stable complexes that it can form with different metallic cations such as Cu^2+^, Co^2+^, Zn^2+^, and Mn^2+^ [1,5,6], plays a key role in our understanding of the catalytic propensity of histidine.

### Abiotic synthesis of adenine and histidine

Adenine was the first imidazole-bearing molecule synthetized under abiotic conditions from an aqueous solution of ammonium cyanide (NH_4_CN) [7]. Since then, many other purines have also been synthesized in prebiotic simulations, including guanine and xanthine [8], hypoxanthine [9], 8-hydroxymethyladenine [10], and 2,6-diaminopurine [11]. Outstandingly, the imidazole group, as well as its 2-methyl derivative were obtained by Oró et al. [12] using glyoxal, aldehydes and ammonia as precursors.

**Fig 2:**
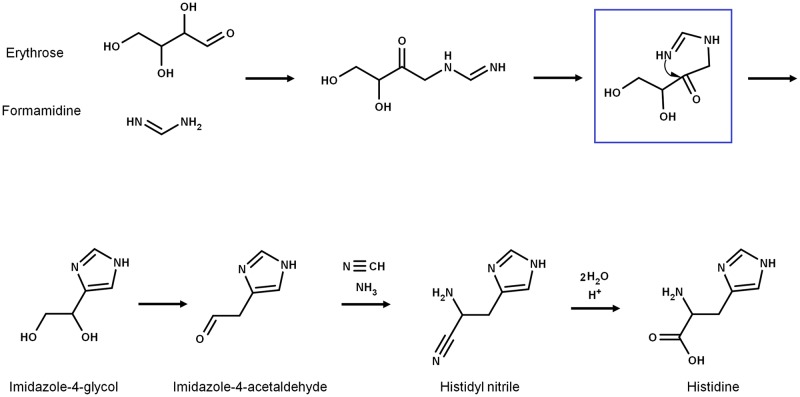
Abiotic synthesis of histidine. The synthesis was proposed by [13,14] and occurs via Strecker synthesis. As shown in the upper part of the scheme, an Amadori rearrangement gives place to the histidine imidazole moiety [cf. ref 17].

The first attempt to synthesize histidine under possible prebiotic conditions was reported by Shen et al. [13,14]. In the proposed reaction, D-erythrose and formamidine are condensed, leading to imidazole-4-acetaldehyde, which then forms histidine via a Strecker synthesis with a relatively high yield of 3.5% based on the ratio of His/erythrose (Fig 2). As argued below, a detailed examination of this reaction suggests that the conditions of synthesis are prebiotically unrealistic, and that it is unlikely it took place in the primitive Earth.

Even though the *a priori* selection of the D-enantiomer of erythrose is irrelevant since the stereocenters do not partake in the synthesis, and the reaction would work if other 4-carbon aldoses were used as precursors, D-erythrose and D-threose are minor, unstable products of the formose reaction [15], and therefore could not have been available in the concentration required for the reaction reported by Shen et al. [13,14] to take place. In addition, formamidine is a labile compound that is rapidly hydrolyzed into formic acid and ammonia [16], which also makes it very difficult to achieve the concentration required for the proposed reaction (0.3 M). As concluded elsewhere, all this data strongly hinders the prebiotic significance of this synthesis [17].

Nevertheless, it is quite interesting that in the reaction mechanism proposed by Shen et al. [13,14] the formation of the imidazole group is the product of an Amadori rearrangement (Fig 2), which is also the mechanism involved in the synthesis of the imidazole moiety of imidazole glycerol phosphate (IGP) by the IGP synthase (HisHF) enzyme during histidine biosynthesis. However, as argued elsewhere [17], all the evidence suggests that there is no direct evolutionary connection between the abiotic synthesis reported by Shen et al. [13,14] and the extant histidine anabolism.

As of today, no imidazole-bearing amino acid has been produced in Miller-Urey-type experiments or in other type of laboratory simulations, and the search of histidine and its degradation products in carbonaceous chondrites has also yielded negative results. These results suggest that histidine as such may have not been present in the prebiotic environment [17–19] but, as argued here, may be in fact a product of very early biological evolution.

### Role of imidazolides in the evolution of catalysis

Although it has been shown that clays, metallic cations, and relatively simple compounds like proline and other amino acids can affect the rates of chemical reactions under possible primordial conditions, the emergence of biological catalysis remains one of the key questions in modern biology.

The discovery of the catalytic properties of RNA [20,21] provided support for the RNA world proposal, where it is assumed that RNA molecules played a key role in both heredity and catalysis during the very early stages of biological evolution.

Although how the RNA world evolved from the prebiotic environment remains an open issue, there have been advances in our understanding of the role that imidazole and imidazole-derivatives could have played during this evolutionary stage. For instance, it has been demonstrated that 2-aminoimidazole can act as an activating group for non-enzymatic RNA copying by a mechanism involving an imidazolium-bridged dinucleotide intermediate at pH = 7 [22]. Unsubstituted imidazole may have also played a role during the RNA world, as suggested by the demonstration that RNA cleavage can be catalyzed by imidazole in buffer [23]. The unsubstituted imidazole group can also act as a cofactor for the hepatitis delta virus (HDV) self-cleaving ribozyme [24]. The reaction mechanism of this ribozyme involves a nucleophilic attack of the 2′-OH on the phosphorus in the phosphodiester backbone, where the catalytic cytosine accepts the proton from the attacking 2′-hydroxyl group. As shown by Perrotta et al. [24], the incubation with unsubstituted imidazole buffer (200 mM, pH 7.4) restores the catalytic activity of a mutated HDV ribozyme. This shows that imidazole by itself can act as a co-catalyst in reactions involving RNA molecules, which could be of key significance in an RNA world scenario, and of considerable significance in our understanding of the early evolution of biological catalysis.

The catalytic activity of His-containing di- and tri-peptides has also been investigated. Histidyl-histidine (His-His) catalyzes the dephosphorylation of deoxyribonucleoside monophosphate, the hydrolysis of oligo (A)_12_, and the oligomerization of 2',3'-cAMP under cyclic wet-dry reaction conditions [25]. More recent studies have shown that seryl-histidine (Ser-His) catalyzes the oligomerization of trimers of imidazole-activated nucleotides [26,27], and catalyze peptide bond formation between two amino acids, an activity also found in seryl-histidyl-glycine (Ser-His-Gly) [28,29].

### Evolution of enzyme catalytic activity from an RNA world

Although there have been alternative proposals to explain the origin of ribosome-mediated protein synthesis based on the simultaneous appearance of both RNA and proteins [30,31], it can also be argued that the ribosome first appeared as an evolutionary outcome of the interaction of prebiotically synthesized amino acids with catalytic RNA molecules. How enzymes first evolved remains an open question, but it is reasonable to assume that their active sites must be one of their most conserved portions. However, if histidine was absent in the primitive Earth, how can its ample distribution in the active sites of enzymes be explained?

In what may be the first attempt to explain the origin of histidine biosynthesis, over 40 years ago Harold White III correlated the start of the histidine biosynthetic pathway, which involves a condensation reaction between ATP and the sugar phosphoribosyl pyrophosphate (PRPP), with the possibility of an ancient metabolism mediated by nucleic acid enzymes [32]. In his evolutionary biochemistry approach, White proposed that histidine was, in fact, the molecular vestige of an ancient catalytic nucleotide that was part of the RNA world [32,33]. According to White, this possibility is reinforced by a) the well-known fact that the histidine anabolic pathway is connected with the *de novo* synthesis of purines by the usage of PRPP and the 5-aminoimidazole-4-carboxamide ribonucleotide (AICAR) intermediate; b) His is the only known amino acid with a ribonucleotide-starting biosynthesis; and c) histidine is the only imidazole-bearing amino acid.

**Fig 3:**
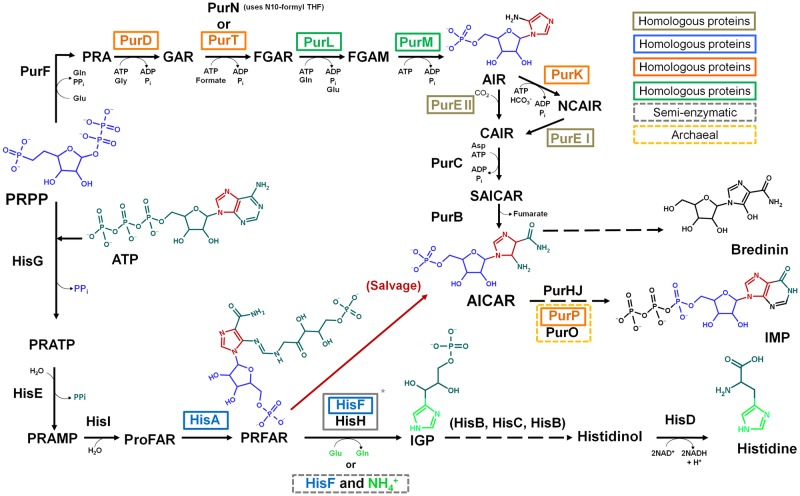
Pathways for the biosynthesis of histidine (bottom) and purines (top). Both routes start with PRPP and are connected by AICAR. The enzymes for each step are depicted. The enzyme name’s color and the boxes indicate homologous proteins within pathways as well as the semi-enzymatic reaction. It is evident how the imidazole group of the ATP molecule along with the pentose of PRPP become part of AICAR, and goes into the purine synthesis, while the imidazole in IGP forms independently. The red arrow indicates the salvage pathway in which AICAR is recycled into the de novo synthesis of purines. The dashed arrows indicate two or more steps in the route, and for simplicity the cofactors and coenzymes used in these steps have been omitted. *The complex formed by the HisF and HisH proteins is named imidazole glycerol phosphate synthase (IGPS). The full names of the most relevant metabolites and enzymes are given in the text.

While it is true that histidine is the only biological amino acid with an imidazole side chain, the other two issues merit a reexamination. As underlined by White [32], during the first step of the histidine biosynthesis, PRPP, whose synthesis in prebiotic conditions has been recently reported by Akouche et al. [34], is condensed with a molecule of ATP to form N’5- phosphoribosyl -ATP (PRATP) (Fig 3). Quite surprisingly, however, the imidazole component of the ATP does not partake in the biosynthesis of the imidazole group of histidine. Instead, the histidine’s imidazole moiety is biosynthesized *de novo* by a mechanism completely different from that of purines.

As shown on Fig 3, histidine- and purine biosyntheses are indeed connected by the imidazole intermediate AICAR, which is formed independently in the two pathways. In the purine route it is formed from succinyl-AICAR (SAICAR), and from N'-((5'-phosphoribulosyl) formimino)-5-aminoimidazole-4-carboxamide-ribonucleotide (PRFAR) in the histidine pathway, but it does not play any further role in the subsequent steps of the histidine anabolism. Instead, it is recycled directly into the purine pathway, where it is used as a substrate by the bifunctional enzyme AICAR transfromylase/inosine monophosphate (IMP) cyclo-hydrolase (PurHJ) [EC: 2.1.2.3/EC: 3.5.4.10], that catalyzes the final two steps in the route and synthesizes IMP (Fig 3). We suggest that the incorporation of AICAR from the histidine biosynthetic route into the *de novo* synthesis of purines can be best understood as an intracellular salvage pathway (Fig 3), in which the imidazole compound is used to supply the requirements of purine synthesis.

### Biosynthesis of the imidazole group in histidine and purines

In the histidine biosynthetic pathway, the enzyme imidazole glycerol phosphate synthase (IGPS) [EC: 2.4.2.-/EC:4.1.3.-] catalyzes the conversion of PRFAR and glutamine to imidazole glycerol phosphate (IGP), AICAR, and glutamate [35]. As mentioned above, AICAR is discarded. In other words, in what appears to be a rather convoluted, non-parsimonious route, the histidine’s imidazole moiety is synthesized *de novo* as part of IGP, while AICAR is recycled into purine synthesis.

**Fig 4:**
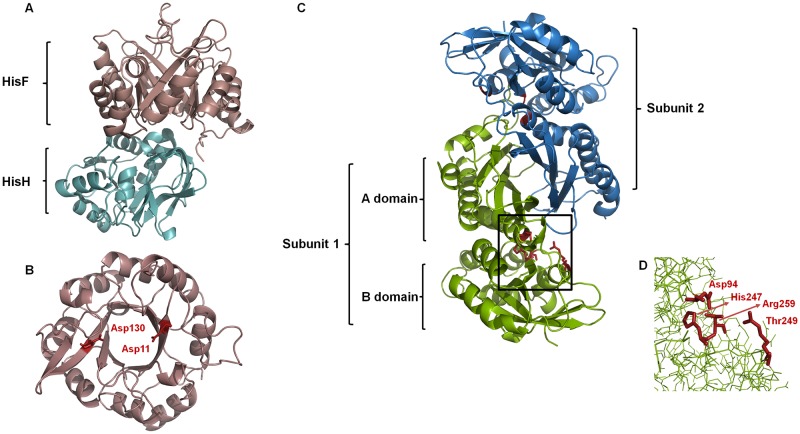
Crystallographic structures of HisF and PurM. (A) Structure of the imidazole glycerol phosphate synthase (IGPS) of *Thermotoga maritima* (PDB:1GPW). (B) Structure of the cyclase (HisF) subunit of *Thermotoga maritima* (PDB: 1THF). Catalytic residues (Asp11 and Asp130) are indicated. (C) Structure of the phosphoribosylformylglycinamidine cyclo-ligase (PurM) of *Escherichia coli* (PDB: 1CLI). (D) Proposed active site for PurM, which comprises the residues Asp94, His247, Thr249, and Arg259.

**Fig 5:**
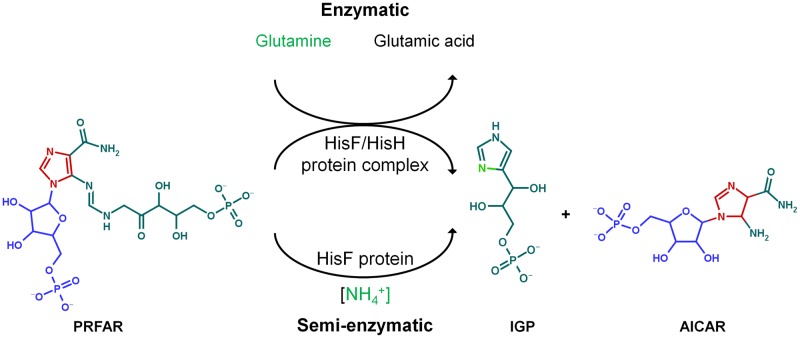
Enzymatic and semi-enzymatic synthesis of imidazole glycerol phosphate (IGP). The enzymatic branch needs both HisF and HisH proteins. HisH transfers nitrogen from glutamine, forming glutamate. In contrast, the semi-enzymatic branch only requires the HisF protein, since the high concentration of NH_4_^+^ in the medium can replace the transferase activity of HisH. In both cases, IGP and AICAR are formed (modified from ref. [41]).

The bacterial IGPS enzyme is a heterodimer formed by the *hisH* and *hisF* gene products (Fig 4A), where *hisH* encodes a glutamine amidotransferase (GATase type I) subunit [EC: 2.4.2.-], and *hisF* encodes a cyclase subunit [EC:4.1.3.-] that adopts the (β/α)_8_-barrel fold [36,37] (Fig 4B). Binding of PRFAR to the HisF active site stimulates the glutaminase activity in the HisH enzyme, revealing an allosteric regulation [38]. The synthesis of IGP can also take place *in vitro* in the absence of the HisH protein under a high concentration of NH_4_^+^ [39]. This phenomenon was also shown to occur *in vivo* in the gammaproteobacteria *Klebsiella pneumoniae*, where the *hisH* gene was mutated but the reaction was restored in high concentrations of NH_4_^+^ [40] (Fig 5). As argued by Lazcano and Miller [41], the fact that the pathway can take place in the absence of HisH suggests that a simpler pathway with lesser number of enzymes may have existed in the distant past. This provides an example of the semi-enzymatic origin of metabolic pathways, in which a primitive biosynthetic route could have exploited a favorable chemical environment as well as available enzymes (Fig 5) [41].

As summarized in Fig 3, the ATP-dependent enzyme phosphoribosylformylglycinamidine cyclo-ligase [EC:6.3.3.1] (PurM) synthesizes 1-(5'-Phosphoribosyl)-5-aminoimidazole (AIR) from 1-(5'-phosphoribosyl)-N-formylglycinamidine (FGAM). This enzyme is present in all purine biosynthetic pathway variations found in the three domains of life. Structurally, each PurM monomer is divided into two domains, an A- or N-terminal domain, and a B- or C-terminal domain (Fig 4c) [42]. The active enzyme is a homodimer formed by the interaction of two four-stranded β-sheets (one from each subunit) forming an eight-stranded β-barrel-like structure (Fig 4C) [42]. AIR is then enzymatically processed into SAICAR, which is the substrate of the enzyme adenylosuccinate lyase [EC:4.3.2.2] (PurB), that catalyzes the synthesis of AICAR [43].

In summary, HisF and PurM are not homologues but they catalyze the biosyntheses of the imidazole motif in the histidine and purine pathways (Fig 3) and do so by completely different mechanisms. The presence of two different independent syntheses of imidazole intermediates in highly conserved, widely distributed routes is rather intriguing and becomes an interesting question from an evolutionary perspective. How can the existence of such biochemically redundant enzymatic complexes in highly connected, early pathways be understood? Here we analyze the active sites of both enzymes and the reactions they catalyze, and propose an explanation for their polyphyletic origin.

## Materials and methods

### Sequence analysis

The sequences of bacterial HisF (*Escherichia coli* K-12 MG1655: b2025), and PurM (*Escherichia coli* K-12 MG1655: b2499) proteins were retrieved from the Kyoto Encyclopedia of Genes and Genomes (KEGG) [44]. Pairwise sequence alignment was performed using the Needleman-Wunsch algorithm as implemented in the EMBOSS Needle program of the European Molecular Biology Open Software Suite [45].

### Structural analysis

Visualization and analysis of protein structures were performed using the PyMol package [46]. Structure comparisons were performed using the FATCAT algorithm [47] included in the protein comparison tool of the Protein Data Bank (PDB) web server. DaliLite v3 was used to search for structural related proteins [48].

### Structure-based dendogram construction

The available crystal structures of the HisF, PurM, and their paralogues were retrieved from the PDB. A total of 38 structures available as of January 2018 (S1 Table) were analyzed following a previously described procedure [49], only structures with no bounded ligands and no reported mutations were considered in this work. To avoid redundancy, for each protein only the structures with the best resolution were selected if two or more of them were available for the same species. Pairwise structural comparisons were performed using the Secondary Structure Matching (SSM) program of the European Protein Data Bank (PDBe) webserver [50]. The structural alignment score (SAS) for each protein-protein comparison was calculated following the formula: (RMSD x 1000)/number of aligned residues [51]. Geometric distance values were calculated using the SAS and then transformed into evolutionary distances using the FITCH program of the PHYLIP package [52]. Finally, dendograms were constructed using the DRAWTREE program included in PHYLIP. Dendograms were visualized and edited using FigTree version 3.2 [53].

## Results

### Imidazole-biosynthetic enzymes HisF and PurM: A case of evolutionary convergence

**Fig 6:**
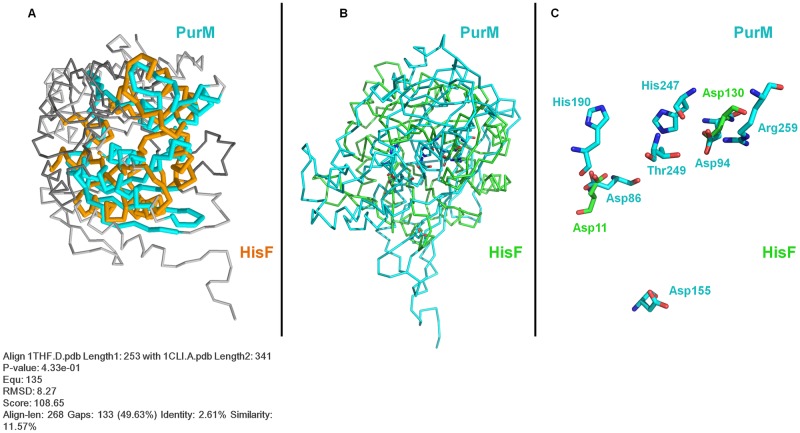
Structural analysis of the HisF and PurM proteins. (A) Structural alignment of HisF from *Thermotoga maritima* (PDB: 1THF) and PurM from *Escherichia coli* (PDB: 1CLI) using the FATCAT algorithm (B) Structural alignment of the active sites of HisF and PurM using the *align* command of PyMol. (C) Catalytic residues that comprise the active sites of HisF and PurM isolated from the alignment in B.

**Fig 7:**
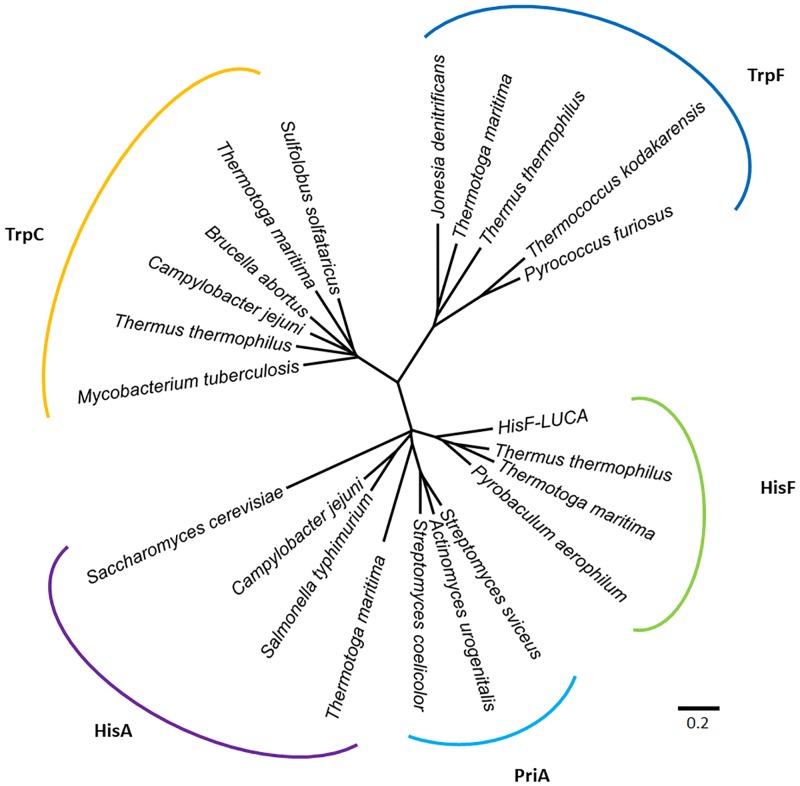
Structural phylogeny of HisF and its homologues. The unrooted tree shows the evolutionary relationships between the different HisF protein paralogs. Of particular interest is the position of the PriA branch, which suggests that this protein arose after diversification of HisA and TrpF. The tree was constructed from comparison of tertiary structures using the Fitch-Margoliash algorithm included in the PHYLIP package [52].

The *E*. *coli* PurM (354 aa) and HisF (258 aa) proteins share only 12.2% of overall sequence identity (21.4% similarity) (S1 Fig). When the crystallographic structures of the two proteins are superimposed, the root-mean-square deviation (RMSD) of the α-carbons is 8.27 Å (Fig 6A). This very high RMSD value reflects of the very different domain architectures of the two enzymes. As noted above, HisF has the (β/α)_8_-barrel fold and belongs to the ribulose-phoshate binding barrel superfamily, while PurM adopts a completely different fold and is part of the PurM superfamily. Considering these structural differences, it can be readily concluded that these are different enzymes with different evolutionary histories that share no common ancestor. The evolutionary relationship between HisF and its structural homologues is depicted in the structural phylogeny shown in Fig 7. The branches more closely related to HisF are those corresponding to the enzyme N’-[(5′-phosphoribosyl) formimino]-5-aminoimidazole-4-carboxamide ribonucleotide (ProFAR) isomerase [EC:5.3.1.16] (HisA). It has been shown that HisF and HisA share a similar internal organization that can be explained as two paralogous modules half the size of the extant sequences [54]. An ancestral module duplicated and gave rise to the ancestral *hisA* gene which, in turn, underwent another duplication and fused, giving rise to the *hisF* gene [35,54,55]. This shared evolutionary history explains the closeness between the branches.

Interestingly, HisA catalyzes an Amadori rearrangement that isomerizes the aminoaldose motif in ProFAR into the aminoketose PRFAR. The same chemical rearrangement is responsible for the imidazole ring closure by HisF, and also occurs in the reaction catalyzed by the tryptophan biosynthetic enzyme phosphoribosylanthranilate isomerase [EC:5.3.1.24] (TrpF), which transforms N-(5-phospho-beta-D-ribosyl) anthranilate (PRAn) into 1-(2-carboxyphenylamino)-1-deoxy-D-ribulose 5-phosphate (CdRP) [56,57]

Once synthesized, CdRP is then processed by the enzyme indole-3-glycerol phosphate synthase [EC:4.1.1.48] (TrpC), which catalyzes the formation of 1-C-(indol-3-yl) glycerol 3-phosphate (rCdRP), CO_2_, and H_2_O. Both TrpF and TrpC are homologues to the HisF/HisA pair, and their evolutionary relationship is shown in Fig 7. Although a prebiotic synthesis of tryptophan has been reported [58], it is likely that this amino acid is a latecomer in evolution [59]. This implies that, in fact, the HisF/HisA pair is more ancient than the TrpF and TrpC proteins. This assumed antiquity is concordant with the key catalytic role played by histidine in many extant enzymes (see above).

PriA is a bisubstrate enzyme capable of catalyzing the isomeration, also via an Amadori rearrangement, of both ProFAR and PRAn into the corresponding aminoketoses. It was first described in *Streptomyces coelicolor* and *Mycobacterium tuberculosis* [60] and, as can be seen in the tree, its branch is projected from the HisA branch, suggesting that PriA evolved from HisA (Fig 7). These results confirm the antiquity of the HisA/HisF pair respect to PriA, and support previous conclusions of a close evolutionary relationship between PriA and HisA [61].

On the other hand, PurM is part of the PurM superfamily, and it is homologous to phosphoribosylformylglycinamidine synthase [EC: 6.3.5.3] (PurL), the enzyme which catalyzes the previous step in the *de novo* synthesis of purines. Both enzymes share structural domains and catalyze biochemical equivalent processes using a very similar ATP-dependent reaction mechanism [43]. The available evidence suggest that PurL is the outcome of a gene duplication followed by a fusion event [62], confirming the role of gene duplication events in the early evolution of metabolic pathways [63].

**Fig 8:**
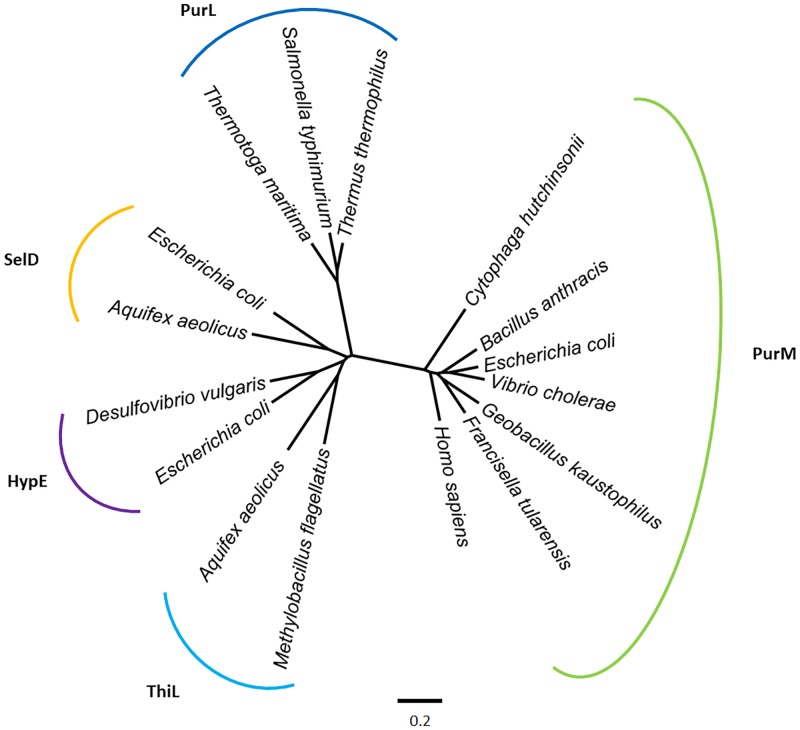
Structural phylogeny of PurM and its homologues. The unrooted tree shows the relationships among the different paralogs of the protein PurM. The tree was constructed from comparison of tertiary structures using the Fitch-Margoliash algorithm included in the PHYLIP package [52].

The search for other structural homologues of PurM identified several proteins of the PurM superfamily, such as the Ni-Fe hydrogenase maturation protein (HypE), selenophosphate synthetase [EC: 2.7.9.3] (SelD), and thiamin phosphate kinase [EC: 2.7.4.16] (ThiL). The evolutionary relationship between all these enzymes is depicted in Fig 8.

Thus, the HisA/HisF pair are homologous proteins that catalyze similar reactions using the same protein scaffold. The same occurs in the PurL/PurM pair. However, the Hisa/HisF and the PurL/PurM pairs are not homologous. In fact, it is well known that analogous catalytic functions can be present in two or more unrelated protein folds. In other words, similar or even identical catalytic functions can appear independently more than once during the evolutionary process, i.e. they are the outcome of convergent evolution [64,65]. As argued here, this is the case of the HisA/HisF and PurL/PurM pairs.

### Active sites and reaction mechanisms of HisF and PurM

There are several types of convergent evolution, including enzymes with similar protein folds that catalyze the identical or similar reactions, as well as convergent evolution of different protein folds catalyzing chemically equivalent reactions on similar or identical substrates, i.e. functional convergent evolution [66].

One of the best-known examples of evolutionary functional convergence at a biochemical level is the case of the subtilisin-like and chymotrypsin-like families of serine proteases. These enzymes adopt different three-dimensional structures, but their active sites evolved independently and resulted in the same catalytic amino acids (the Ser-His-Asp triad), as well as a very similar catalytic mechanism for the hydrolysis of the peptide bond [67,68]. As described below, we have searched for this kind of convergence in the HisF and PurM enzymes.

Biochemical studies of HisF of *Thermotoga maritima* have shown that the essential residues for catalytic activity are Asp11 and Asp130 (Fig 4B). It has been proposed that the carboxylate groups of both aspartate residues are involved in general acid/base catalysis [69]. On the other hand, based on sequence conservation between several PurM enzymes, residues Asp94, His247, Thr249, Arg259 (Fig 4D) have been identified as key components of the active site for the PurM of *Escherichia coli*, where these residues may be involved in general acid and base catalysts, as well as in the coordination of metal ions [42]. As shown in Fig 6C, the use of the *align* command of PyMol to superimpose the active sites of HisF and PurM demonstrates that no spatial correspondence exists between them (Fig 6C). Our comparison reveals that the similarities between the two enzymes are limited to the structural correspondence between the Asp130 of HisF and the Asp94 of PurM and, to a certain extent, to the proximity between the Asp11 of HisF and Asp86 of PurM. Howerver, the rest of the active site residues of PurM have no structural counterpart in the HisF structure. This implies that the architecture of the active sites of HisF and PurM is completely different, and thus it can be safely concluded that their biochemical activities are not due to the convergence of their catalytic sites.

**Fig 9:**
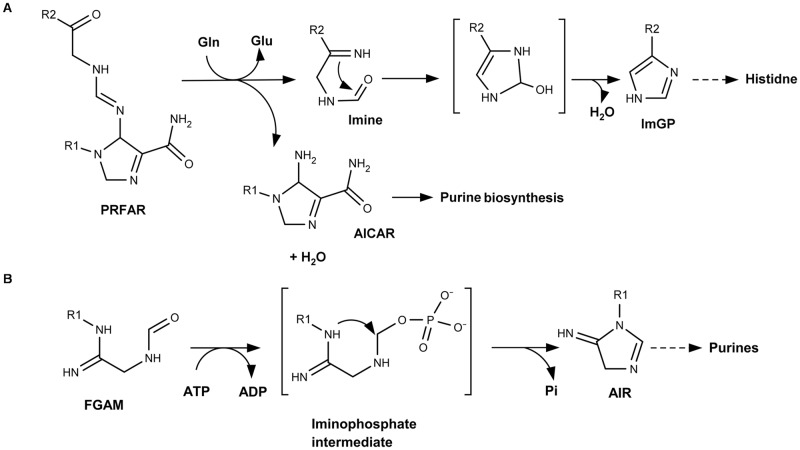
Proposed reaction schemes for HisF and PurM. The reaction schemes are based on [42, 69]. The nucleophilic attack of amino to the carbonyl group is depicted. R1: ribose phosphate; R2: triose phosphate.

Moreover, analysis of the reaction mechanisms proposed for each of the imidazole-synthesizing enzymes [42,69] demonstrates their profound differences, and allows to describe the specific details of the cyclization of the imidazole motif in each case. As noted above, the HisF enzyme catalyzes an Amadori rearrangement that is responsible for the formatiom of the imidazole ring, where the cyclization occurs after the hydrolysis of AICAR from the substrate PRFAR. This exposes a carbonyl group that can then react with the amino group of the same substrate (Fig 9). On the other hand, PurM uses one ATP molecule to activate a carbonyl group present in the FGAM structure, generating an iminophosphate intermediate. Immediately after this activation, an amino group makes a nucleophilic attack towards the previously-activated carbonyl group, leading to the cyclization of the imidazole ring. As depicted in Fig 9, the imidazole cyclization in both enzymes occurs by means of a nucleophilic attack of a nitrogen towards a carbonyl group. However, the overall mechanisms are completely different, with HisF catalyzing an Amadori rearrangement, and PurM catalyzing a strictly ATP-dependent reaction. In other words, the two enzymes are not homologous and the biosyntheses of the two imidazole groups proceed via different mechanisms.

## Discussion

### Evolutionary convergence in biochemistry

An example of biochemical redundancy is found in the biosyntheses of lysine, where three different anabolic pathways have been reported, i.e., the diaminopimelic acid pathway (DAP), the α-aminoadipic acid route (AAA) [70,71], and the recently-discovered TK0280-TK0283 variant found in *Thermococcus kodakarensis* [72].

Evolutionary functional convergence has also been described in the families of kinases that catalyze the phosphorylation of sugars, specifically in the hexokinase, ribokinase and galactokinase families, where two types of convergence can be recognized [66]. In the first case, a common trait originated independently several times in the same protein fold, such as the specificity to phosphorylate glucose, which clearly appeared more than once within the hexokinase family. In the second case, different protein structures convergently evolve to the same catalytic function, as occurred with the use of fructose as a substrate, which appeared independently in both the ribokinase and the hexokinase enzyme families [66]. The case of the imidazole-synthesizing HisF and PurM enzymes discussed here not only represents another example of this second kind of convergent evolution, but also underlines the role of redundancy in the biochemical syntheses of the imidazole group. As summarized below, this is best understood in terms of the extraordinary biological significance of this chemical moiety.

### Evolution of two different imidazole-synthesizing enzymatic systems

As described above, histidine biosynthesis starts with ATP. This ribonucleotide is condensed with PRPP to form PRATP (Fig 3), which is then enzymatically processed to form AICAR. As underlined above, rather surprisingly the imidazole moiety in AICAR (which is derived from ATP) is not used to form histidine. Instead, a new imidazole moiety is biosynthesized and becomes part of histidine. The obvious question is why the available imidazole moiety already present in AICAR is not used for such task. As explained below, we suggest that the answer is found in the differences between the enhanced catalytic properties of the imidazole moiety of histidine as compared to that of purines.

Adenine has a well-known ability to coordinate a series of metal ions, including Cu^2+^ and Ag^+^ [73]. It has been shown that artificially constructed adenine-containing matrices can coordinate such metals and exhibit catalytic activities, including hydrolysis of ester substrates such as *p*-nitrophenyl phosphate (*p*NPP), bis-(*p*-nitrophenyl) phosphate (bNPP) and 2-hydroxypropyl-*p*-nitrophenyl phosphate (hNPP), as well as the hydrolysis of 2’-3’-cAMP [73]. Metal binding is equally significant for the catalytic capabilities of RNA, together with the highly reactive ribose 2'-hydroxyl group and the tertiary structure that these molecules adopt. Although it has been demonstrated that the catalytic activity of the ribosome is dependent on an adenine base [74,75], the reaction mechanism involves a proton abstraction by the N3 of the adenine with no direct participation of the imidazole ring, which, as of today, has not been shown to participate in any of the known catalytic activities of RNA.

These results are explained by the fact that in both N6 ribosyl adenine and histidine the imidazole rings remain unbound, which leaves them free to react [76,77]. Nevertheless, this could change if the covalent bond that joins the ribose with the base is changed from position N9 to the position N6. In fact, as demonstrated by Maurel and Ninio [76], N6 ribosyl adenine can accelerate the hydrolysis of *p*-nitrophenyl acetate (PNPA) in solution at pH 7.7, a reaction that neither adenosine nor AMP can catalyze. The rate at which N6 ribosyl adenine accelerates the reaction is comparable to that observed when histidine is used as catalyst.

In sharp contrast with histidine, the β-glycosidic bond between the N9 of the imidazole ring in purine nucleotides and the C1' of the ribose blocks the otherwise acidic N9 atom, completely inhibiting the catalytic capabilities of its imidazole moiety. In other words, if a molecule such as N6-ribosyl adenine would be incorporated into RNA, we would expect that its imidazole ring would exhibit catalytic activities. This possibility is amenable to experimental analysis.

It is worth noting that in sharp contrast with pyrimidines, in the *de novo* synthesis of purines this β-N9-glycosidic bond is formed in the very first step of the anabolic pathway, and along the entire anabolic process the purine biosynthetic intermediates are ribotide derivatives, constraining the catalytic capabilities of the imidazole moiety from the start of the route.

We surmise that these structural changes in the imidazole moieties account for the major differences in the catalytic properties between histidine and adenine, and were probably the critical issue in the emergence of histidine biosynthesis, as well as an important selection pressure for the evolution of two non-homologous routes for the synthesis of imidazole group.

### Evolution of histidine and purine biosynthesis

The phylogenetic distribution of the enzymes involved in histidine and purine *de novo* biosyntheses shows that both pathways are highly conserved through the three domains of life, suggesting that they originated and were well established before the diversification of the Last Universal Common Ancestor (LUCA) into major kingdoms [78]. Taking into consideration the functional convergence of the enzymes that catalyze imidazole syntheses, the two pathways must have evolved independently during very early stages of protein evolution, using the same pentose derivative as precursor, and later on became connected through AICAR, which as underlined above can be considered as a mere intracellular salvage step. Given the key role of purines in RNA properties, including catalysis, it is reasonable to conclude that purine biosynthesis appeared before histidine anabolism.

Sequence analysis of the enzymes that catalyze the different steps in the pathways provide information of their evolution. Considering the number of paralogous genes that both HisF and PurM have within their respective routes (Fig 3), it is reasonable to hypothesize that the present enzymatic activities of these two proteins evolved from ancestral enzymes with less specificity for substrates. For instance, in addition to the PurL/PurM pair, the archeal *purD*, *purT*, *purK* and *purP* genes are also homologous [43]. This suggests the existence of at least two ancestral enzymes with relaxed substrate specificity, that could catalyze all the corresponding activities of the modern paralogous enzymes. This possibility is certainly consistent with the idea of a patchwork assembly of the routes [79]. In histidine biosynthesis, the experimental evidence indicating that the synthesis of IGP can take place, both *in vivo* and *in vitro*, in the absence of the HisH protein [40], as well as the likely existence of an ancestral enzyme with both HisA and HisF activities, suggest that primitive histidine biosynthesis was simpler in the early stages of its evolution, during which spontaneous transformations coexisted with enzymes with low substrate specificity catalyzing similar chemical reactions [41]. As summarized in Fig 3, a simpler histidine anabolism can thus be envisioned.

### AICAR has an evolutionary adaptive value by its own

**Fig 10:**
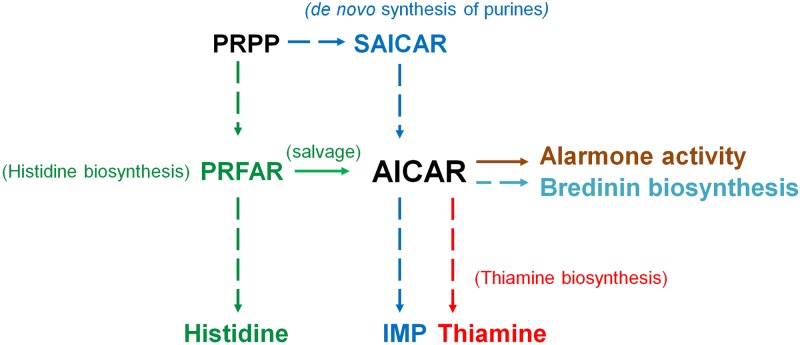
Biological significance of a metabolic intermediary. Scheme showing the roles of the imidazole intermediary AICAR in extant metabolism. From the salvage biosynthesis pathway from histidine to the *de novo* synthesis of purines.

The manifold roles of AICAR in biochemical process go beyond the proposed salvage pathway connecting the histidine and the *de novo* synthesis of purines. For example, it has been shown that AICAR accumulates in *purH* mutants and inhibits the conversion of AIR to the 4-amino-5-hydroxymethyl-2-methylpyrimidine (HMP) moiety of thiamine [80], and that can also decrease total coenzyme A pools inhibiting the activity of the enzyme pantoate-alanine ligase in *Salmonella enterica* [81]. Perhaps more significantly, both AICAR and its triphosphorylated form (5-amino 4-imidazole carboxamide riboside 5’-triphosphate, also known as ZTP) can act by themselves as alarmones in C-1-folate deficiency [82]. AICAR accumulation also leads to histidine auxotrophy and even growth arrest in yeast [83]. AICAR is also the precursor for the antibiotic bredinin (5-hydroxyl-1-beta-D-ribofuranosyl-1H-imidazole-4-carboxamide), which is part of the naturally occurring family of nucleotide antibiotics first isolated from *Eupenicillium brefeldianum* M2166 [84]. This underlines the significance of AICAR, which during evolution has acquired other functions beyond that of an intermediate in purine biosynthesis (Fig 10). This may be seen as a case of exaptation, in which evolution selected AICAR into other roles, with that of alarmone being among the earliest. The fact that both AICAR and ZTP can bind to functional RNA molecules [85], and that 4-amino-5-imidazole carboxamide (AICA) can be synthesized under prebiotic conditions [86,87] may also speak of the antiquity of this molecule.

### Other imidazole syntheses in the cell

In addition to the histidine and purines pathways there are other imidazole-synthesizing routes present in the extant cells, which also underline the key role of imidazole and imidazole-derivatives. All the biological and abiotic forming mechanisms of imidazolides represent a case of redundancy for the synthesis of this chemical group and highlight its catalytic significance.

**Fig 11:**
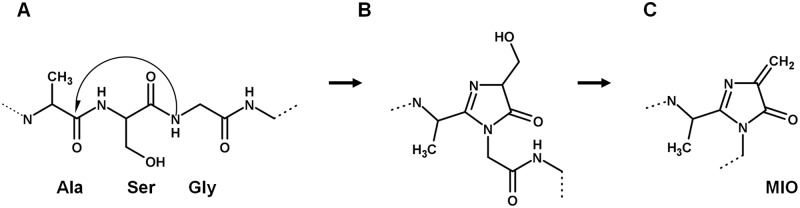
Mechanism of MIO formation. MIO formation via cyclization of an Ala-Ser-Gly- motif. (Modified from ref. [89]). Chemical structures were drawn using MarvinSketch v16.1.4 ChemAxon [94].

Some examples include the anabolic routes of (i) the protein cofactor biotin; (ii) the molecule creatinine; and (iii) the autocatalytically-formed prosthetic group 4-methylideneimidazole-5-one (MIO). Biotin is a vitaminic cofactor that transfers CO_2_ as well as C-2 units, and its imidazole moiety is formed enzymatically by action of the dethiobiotin synthetase [EC:6.3.3.3] (BioD) enzyme. Quite significantly, both creatinine and MIO are imidazole molecules formed non-enzymatically. Creatinine is formed via the spontaneous cyclization of creatine-phosphate [88]. On the other hand, MIO participates as a prosthetic group in the histidine-, tyrosine- and phenylalanine ammonia-lyases (HAL, TAL and PAL, respectively) enzymes, that catalyze the α, β-elimination of ammonia from the corresponding amino acid using MIO as an electrophile, and are structurally homologous to the tyrosine and phenylalanine amininomutases (TAM and PAM), which catalyze the interconversion of a β-amino acid from an α-amino acid using the same MIO cofactor. This imidazole cofactor is formed by condensation of a highly conserved alanine-serine-glycine motif located within the active site of the enzymes and is used as an electrophile [89]. The reaction starts with the attack of the Gly amine to the carbonyl group of Ala, generating a five-membered intermediate, which is then transformed into the MIO cofactor (Fig 11) [90]. Given the well-established fact that Ala, Ser and Gly are three of the most abundant amino acids found in prebiotic experiments [91,92] as well as in carbonaceous chondrites [93] a possible model prebiotic experiment using the Ala-Ser-Gly in order to obtain an imidazolide has recently been proposed [17].

## Conclusions

In contrast to other cases of convergence in biosynthetic pathways such as those reported for polyphyletic caffeine biosyntheses [65], in the different routes for imidazole formation discussed here the final products are not the outcome of enzyme recruitment via a patchwork mechanism, but rather examples of deterministic chemical processes that lead via different processes to the same functional product.

Like all other biological traits, the properties of biochemical pathways have been shaped by their evolutionary history. History, however, is not a mere sequence of chance events. Natural selection can overcome contingent effects to an extent, and chemical and biochemical constraints may have had a greater significance than is normally recognized in shaping metabolic traits. This appears to be the case of the manifold ways in which imidazole moieties are formed in extant cells.

As reviewed here, analysis of metabolic processes and biochemical properties demonstrate that several independent mechanisms of imidazole biosyntheses have evolved. The significance of this chemical group for living organisms is evident as their metabolism became redundant in respect to their syntheses, although the specific reaction mechanisms of each pathway are clearly different. In the purine and histidine biosynthesis, HisF and PurM have different sequences and unrelated three-dimensional structures; however, the two enzymes catalyze analogous reactions that lead to the formation of the imidazole group, representing a case of convergence at a biochemical level. This speaks for the catalytical and structural significance of imidazole and its derivatives.

The two-different enzyme-dependent syntheses of histidine and purines, catalyzed by PurM and HisF, together with the autocatalytic formation of creatinine (from creatine-phosphate) and MIO (from the Ala-Ser-Gly tripeptide) are unique examples of biochemical redundancy that underline for the significance of imidazole derivatives in biological processes. As argued here, the polyphyletic origin of PurM and the HisF is best understood by the enhanced catalytic activity of the histidine imidazole group compared to that of purines, where the β-glycosidic bond between the ribose and the imidazole ring of purines is established from the very start of IMP biosynthesis and results in the inhibition of purine catalytic activities.

The formation of the imidazole motifs in purines and histidine proceeds by means of a nucleophilic attact of a nitrogen atom in an amino group to a carbon atom in the substrate. However, as detailed here, the corresponding imidazole-synthesizing enzymes have different amino acid sequences and different three-dimensional folds, but catalyze chemically equivalent reactions. Thus, in spite of chemical restrictions, considerable degrees of freedom in the sequence space were available during the early stages of metabolic evolution.

## Supporting information

S1 Fig Pairwise alignment of bacterial (*Escherichia coli*) HisF and PurM sequences. The corresponding HisF and PurM sequences of *E*. *coli* were aligned using the Needleman-Wunsch algorithm as implemented in the EMBOSS Needle program of the European Molecular Biology Open Software Suite [45]. The aligment shows 58.5% of gaps with only 12.2% of sequence identity and 21.4% of sequence similarity.
(TIF)

S1 Table Crystallographic structures used for the analysis. Complete list of the crystallographic structures and their PDB codes used to construct the dendograms.
(DOCX)
