# Supplementary figures and images for: Evolutionary convergence in the biosyntheses of the imidazole moieties of histidine and purines
Source: PLoS One. 2018 Apr 26;13(4):e0196349. doi: 10.1371/journal.pone.0196349 (PMC5919458; doi:10.1371/journal.pone.0196349)

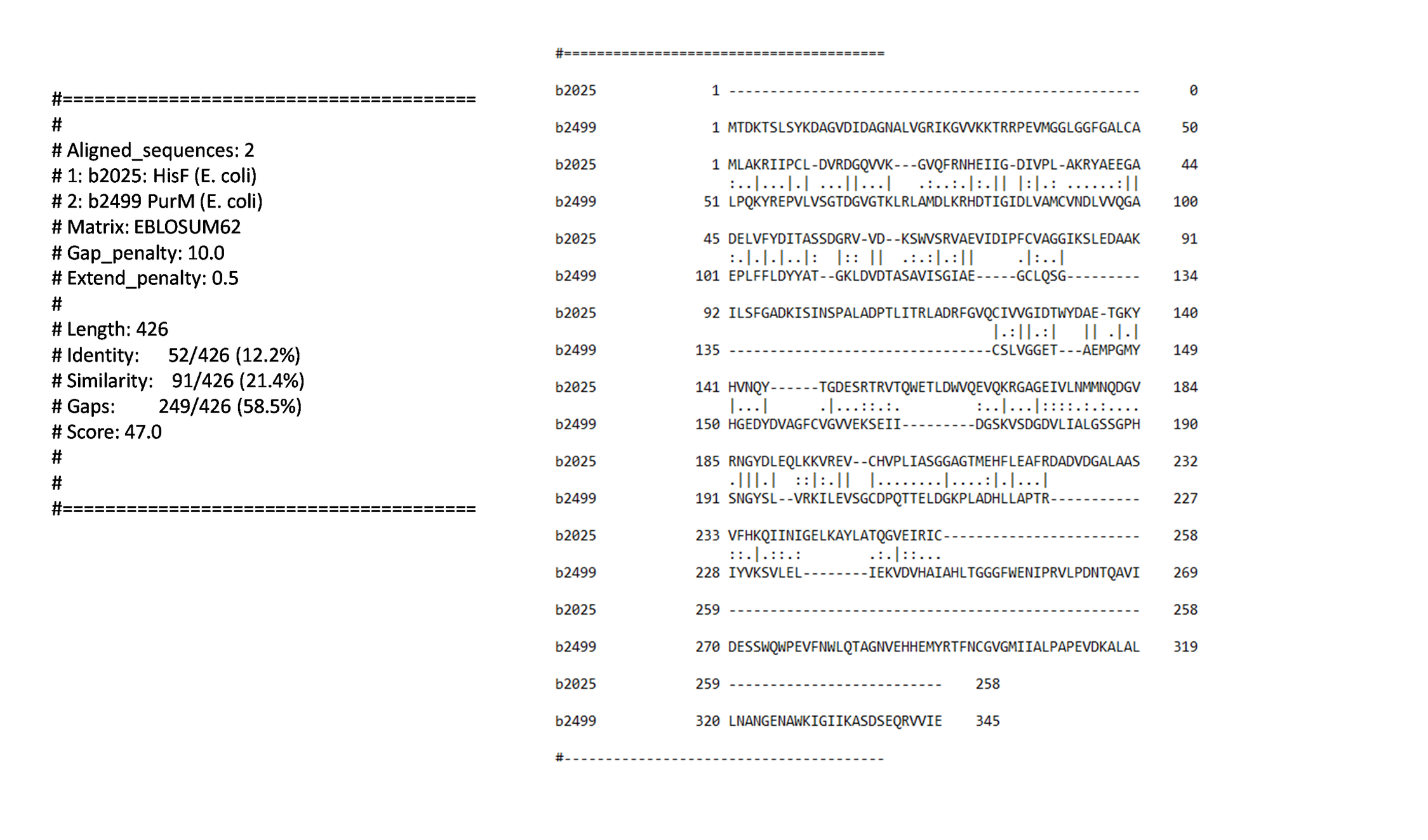

Supplement: S1 Fig — The corresponding HisF and PurM sequences of E. coli were aligned using the Needleman-Wunsch algorithm as implemented in the EMBOSS Needle program of the European Molecular Biology Open Software Suite [45]. The aligment shows 58.5% of gaps with only 12.2% of sequence identity and 21.4% of sequence similarity. (TIF) [file pone.0196349.s001.tif]
