# Supplementary material for: Evolutionary convergence in the biosyntheses of the imidazole moieties of histidine and purines
Source: PLoS One. 2018 Apr 26;13(4):e0196349. doi: 10.1371/journal.pone.0196349 (PMC5919458; doi:10.1371/journal.pone.0196349)
Supplement: S1 Table — Complete list of the crystallographic structures and their PDB codes used to construct the dendograms. (DOCX) [file pone.0196349.s002.docx]

| **PDB code** | **Protein** | **Species** | **Resolution [Å]** |
| --- | --- | --- | --- |
| 3P4E | PurM | *Vibrio cholerae* | 1.77 |
| 2Z01 | PurM | *Geobacillus kaustophilus* | 2.2 |
| 3M84 | PurM | *Francisella tularensis* | 1.699 |
| 2V9Y | PurM | *Homo sapiens* | 2.1 |
| 1CLI | PurM | *Escherichia coli (strain K12)* | 2.5 |
| 2BTU | PurM | *Bacillus anthracis* | 2.31 |
| 3KIZ | PurM | *Cytophaga hutchinsonii (strain ATCC 33406 / NCIMB 9469)* | 1.5 |
| 3VIU | PurL | *Thermus thermophilus (strain HB8 / ATCC 27634 / DSM 579)* | 2.35 |
| 1VK3 | PurL | *Thermotoga maritima (strain ATCC 43589 / MSB8 / DSM 3109 / JCM 10099)* | 2.15 |
| 1T3T | PurL | *Salmonella typhimurium (strain LT2 / SGSC1412 / ATCC 700720)* | 1.9 |
| 1VQV | ThiL | *Aquifex aeolicus (strain VF5)* | 2.65 |
| 3MCQ | ThiL | *Methylobacillus flagellatus (strain KT / ATCC 51484 / DSM 6875)* | 1.91 |
| 2Z1T | HypE | *Desulfovibrio vulgaris (strain Hildenborough / ATCC 29579 / NCIMB 8303)* | 2.6 |
| 2I6R | HypE | *Escherichia coli (strain K12)* | 2.51 |
| 3U0O | SelD | *Escherichia coli (strain K12)* | 2.25 |
| 2ZOD | SelD | *Aquifex aeolicus (strain VF5)* | 1.98 |
| 4EVZ | HisF | Ancestral reconstruction | 1.46 |
| 1H5Y | HisF | *Pyrobaculum aerophilum (strain ATCC 51768 / IM2 / DSM 7523 / JCM 9630 / NBRC 100827)* | 2 |
| 1THF | HisF | *Thermotoga maritima (strain ATCC 43589 / MSB8 / DSM 3109 / JCM 10099)* | 1.45 |
| 1KA9 | HisF | *Thermus thermophilus (strain HB8 / ATCC 27634 / DSM 579)* | 2.3 |
| 5AHE | HisA | *Salmonella typhimurium (strain LT2 / SGSC1412 / ATCC 700720)* | 1.7 |
| 1QO2 | HisA | *Thermotoga maritima (strain ATCC 43589 / MSB8 / DSM 3109 / JCM 10099)* | 1.85 |
| 4GJ1 | HisA | *Campylobacter jejuni subsp. jejuni serotype O:2 (strain ATCC 700819 / NCTC 11168)* | 2.152 |
| 2AGK | HisA | *Saccharomyces cerevisiae (strain ATCC 204508 / S288c)* | 1.3 |
| 1VC4 | TrpC | *Thermus thermophilus* | 1.8 |
| 3QJA | TrpC | *Mycobacterium tuberculosis (strain ATCC 25618 / H37Rv)* | 1.29 |
| 1IGS | TrpC | *Sulfolobus solfataricus (strain ATCC 35092 / DSM 1617 / JCM 11322 / P2)* | 2 |
| 3TSM | TrpC | *Brucella abortus (strain 2308)* | 2.15 |
| 6BMA | TrpC | *Campylobacter jejuni subsp. jejuni serotype O:2 (strain ATCC 700819 / NCTC 11168)* | 1.98 |
| 1I4N | TrpC | *Thermotoga maritima (strain ATCC 43589 / MSB8 / DSM 3109 / JCM 10099)* | 2.5 |
| 4WUI | TrpF | *Jonesia denitrificans (strain ATCC 14870 / DSM 20603 / CIP 55134)* | 1.09 |
| 1NSJ | TrpF | *Thermotoga maritima (strain ATCC 43589 / MSB8 / DSM 3109 / JCM 10099)* | 2 |
| 5LHE | TrpF | *Thermococcus kodakarensis (strain ATCC BAA-918 / JCM 12380 / KOD1)* | 1.85 |
| 1V5X | TrpF | *Thermus thermophilus* | 2 |
| 4AAJ | TrpF | *Pyrococcus furiosus (strain ATCC 43587 / DSM 3638 / JCM 8422 / Vc1)* | 1.75 |
| 1VZW | PriA | *Streptomyces coelicolor (strain ATCC BAA-471 / A3(2) / M145)* | 1.8 |
| 4X2R | PriA | *Actinomyces urogenitalis DSM 15434* | 1.05 |
| 4U28 | PriA | *Streptomyces sviceus ATCC 29083* | 1.33 |
